# Wastewater based environmental surveillance of toxigenic *Vibrio cholerae* in Pakistan

**DOI:** 10.1371/journal.pone.0257414

**Published:** 2021-09-30

**Authors:** Tanzeel Zohra, Aamer Ikram, Muhammad Salman, Afreenish Amir, Asim Saeed, Zurva Ashraf, Abdul Ahad

**Affiliations:** Public Health Laboratories Division, Department of Microbiology, National Institute of Health, Islamabad, Pakistan; Xiamen University - Malaysia Campus: Xiamen University - Malaysia, MALAYSIA

## Abstract

**Background:**

Pakistan has been experiencing intervals of sporadic cases and localized outbreaks in the last two decades. No proper study has been carried out in order to find out the environmental burden of toxigenic *V*. *cholerae* as well as how temporal and environmental factors associated in driving cholera across the country.

**Methods:**

We tested waste water samples from designated national environment surveillance sites in Pakistan with *RT-PCR* assay. Multistage sampling technique were utilized for samples collection and for effective sample processing Bag-Mediated Filtration system, were employed. Results were analysed by district and month wise to understand the geographic distribution and identify the seasonal pattern of *V*. *cholera* detection in Pakistan.

**Results:**

Between May 2019, and February 2020, we obtained and screened 160 samples in 12 districts across Pakistan. Out of 16 sentinel environmental surveillance sites, 15 sites showed positive results against cholera toxigenic gene with mostly lower CT value (mean, 34±2) and have significant difference (p < 0.05). The highest number of positive samples were collected from Sindh in month of November, then in June it is circulating in different districts of Pakistan including four Provinces respectively.

**Conclusion:**

*V*. *cholera* detection do not follow a clear seasonal pattern. However, the poor sanitation problems or temperature and rainfall may potentially influence the frequency and duration of cholera across the country. Occurrence of toxigenic *V*. *cholerae* in the environment samples showed that cholera is endemic, which is an alarming for a potential future cholera outbreaks in the country.

## Introduction

Pakistan is facing a high burden of diverse emerging and re-emerging infectious diseases. Many of them remain undiagnosed and untreated due to weak healthcare system and ineffective regulation [1]. Diarrheal diseases result in 2.1 million deaths annually and given second highest priority for research in particular to infectious diseases. [2]. Diarrheal illnesses were responsible for 15% of the deaths worldwide among children under 5 years of age in 2008 [3]. Pakistan, with 465,000 estimated annual child deaths in 2008, has the fourth highest burden of child mortality in the world, contributing 5% or 1 in every 20 child deaths to the global child mortality pie [4]. Diarrhea results in greatest number of morbidity and mortality which is mainly caused by minimal awareness on hygiene and consumption of contaminated water. Drinking water is contaminated either by human fecal materials or from several warm blooded mammals [5]. Around 79% of the fresh water is not drinkable, and can cause a range of water borne diseases, to which children are at higher risk, especially children of age group <5 years [6]. Overall it has been estimated that 3.4 million people accumulatively are affected worldwide by water borne diseases like acute watery diarrhea, dysentery, hepatitis, and typhoid etc. [7]. In order to limit the disease outbreak, it’s essential to closely monitor and detect the presence of infectious microorganism to substantive evidence for investment in water sanitation and hygiene (WASH). This could not only protect the community’s health but could also help in taking all the necessary measures in terms of management [8].

Cholera is one of the major pathogen that causes water borne outbreaks. The infection is mainly caused by *V*. *cholera* which releases a toxin in the small intestine i.e Cholera toxin. The toxin is encoded by ctxAB genes which caused the epidemic cholera and resulted in the life threatening disease cholera gravis. The type of diarrhea caused is watery in nature which leads to dehydration, shock and ultimately death if left untreated [9]. Cholera attacks the community twice in a year, previously hitting once in the dry season, when there is low river flow, and later on during the fall wet season, when there is heavy rains which result in overflowing of river that leads to floods and destroy sanitation systems in a country [10–12]. According to literature, cholera typically increases from November to January; and from April to May [13–15]. We are also aiming to assess the relative abundance of the toxigenic strains across seasons.

Global trends has shown that cholera has been increased steadily during the course of five years. For instance, a total of 676,651 cases were documented between the years 2000 to 2004. In contrast, up to 2008, a total of 838,315 cases have been diagnosed with cholera as documented by World Health Organization (WHO) [21]. This data clearly shows an increase of diagnosed cases by 24% within 5 years. In 2018, WHO have approximated that 1.3–4.0 million cases were diagnosed with cholera in which 21,000 to 143,000 died globally. The main cause of this outbreak was stated as the shortage of sanitation and potable water facility. Before 1988, cholera was never called a significant cause of diarrhea in Pakistan but now, it has been declared as an endemic disease [16]. Several causes have been discovered which complicate the disease burden estimate including insufficient systemic study, restricted abilities of observational system, deficiency in systematic and methodological studies and travel sanction between the government and healthcare providers as well as the differences in reporting processes [17].

In year 2005, WHO came up with a system, Disease Early Warning System (DEWS).The system was established all over Pakistan in order to initiate prompt investigations alleviate the outbreaks of various diseases by providing proper active surveillance [17]. In year 2005–2009, the system gave its best and was successful in responding to outbreaks which were called as suspected cases of cholera among which 261 were warnings and 46 outbreaks. Following the floods in Pakistan, in year 2010, the ministry of Health confirmed 99 cases of *V*. *Cholera* [18]. Although, the outbreak of cholera is rising steadily in Pakistan but there is no *V*. *Cholera* environment surveillance data is available.

In view of studies from Uganda, Bangladesh and in other countries, it is observed that lake, surface water sources act as environmental reservoirs for pathogenic *V*. *cholerae* responsible for cholera epidemics, as documented in Asia [19–21]. Likewise in 2015, according to a study conducted in Pakistan shows that the rise in cholera cases were due to improper sanitation, unhygienic practices by general public and inaccessibility to safe water [22]. Beside this cholera doesn’t follow a proper seasonal trend and circulating in waste water almost over the year.

Since 2011, there is no comprehensive study has been conducted to described the burden of toxigenic *V*. *cholerae* in environmental samples of Pakistan. However, there is no proper surveillance system is currently working across country even for reporting cholera cases due to limited resources and over all fragile surveillance system of all infectious diseases. Hence an efficient way of tackling infectious pathogens in endemic areas is waste water surveillance [23]. This way, there will be no need for a large population to undergo testing which is not only time consuming but can also be expensive. In order to investigate and detect future pandemics, it has been demanded by the WHO to accumulate all the necessary information on microorganism present in an environment [24].

The urgent need to establish a rapid surveillance system is because the frequency of outbreak is on rise and therefore, it’s essential to follow up on the already existing organisms with in the human population as well as explore the lately discovered pathogens by means of an efficient way such as waste water surveillance based on epidemiology. Therefore, water-based epidemiology (WBE) is an effective tool in order to investigate and detect the presence of pre-existing pathogens such as poliovirus, cholera or typhoid along with the newly emerging infectious agents [25].

**Fig 1:**
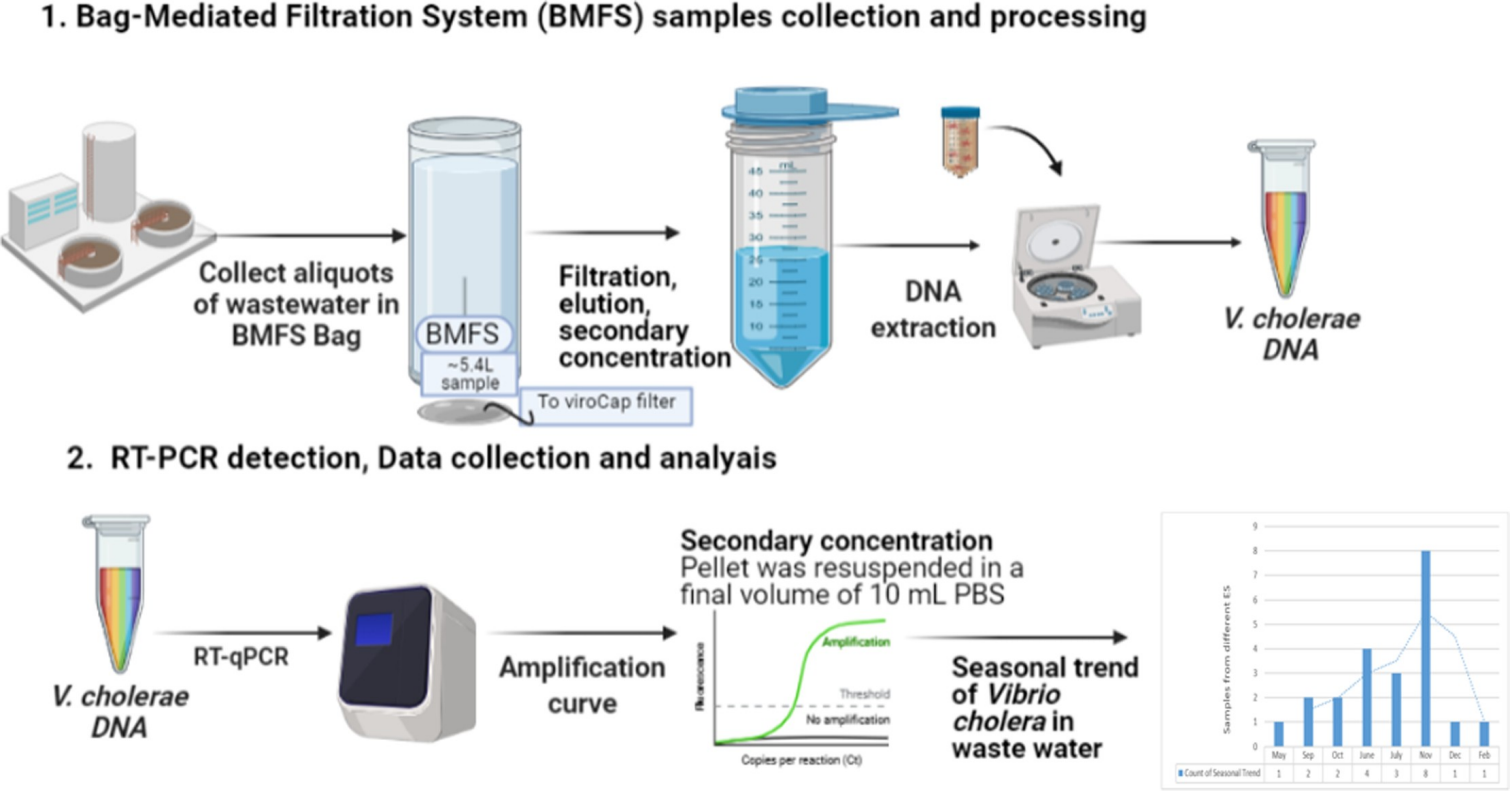
Graphical representation of study conducted.

Cholera attacks the community twice in a year, previously hitting once in the dry season, when there is low river flow, and later on during the fall wet season, when there is heavy rains which result in overflowing of river that leads to floods and destroy sanitation systems in a country [10–12]. According to literature, cholera typically increases from November to January; and from April to May [13–15]. For this we aimed to assess the relative seasonal abundance of toxigenic Vibrio and its association with environmental factors and to find out the annual geographic distribution of toxigenic *V*. *cholerae* across different regions of Pakistan (Fig 1).

## Methodology

### Study design

A prospective study was conducted to find out the frequency of *V*. *cholerae* in wastewater.

### Sampling technique, duration and size

Multistage sampling technique were utilized for samples collection. Samples were collected for a period of 10 months from May 2019 to February 2020, on a samples size, n = 160. Frequency of collected samples were once per month from 16 sites over the period of 10 months. Non probability convenience sampling was adopted to examine the environmental surveillance of *V*. *cholerae* in Pakistan.

### Study sites for toxigenic *V*. *cholerae* Environment Surveillance (ES)

**Fig 2:**
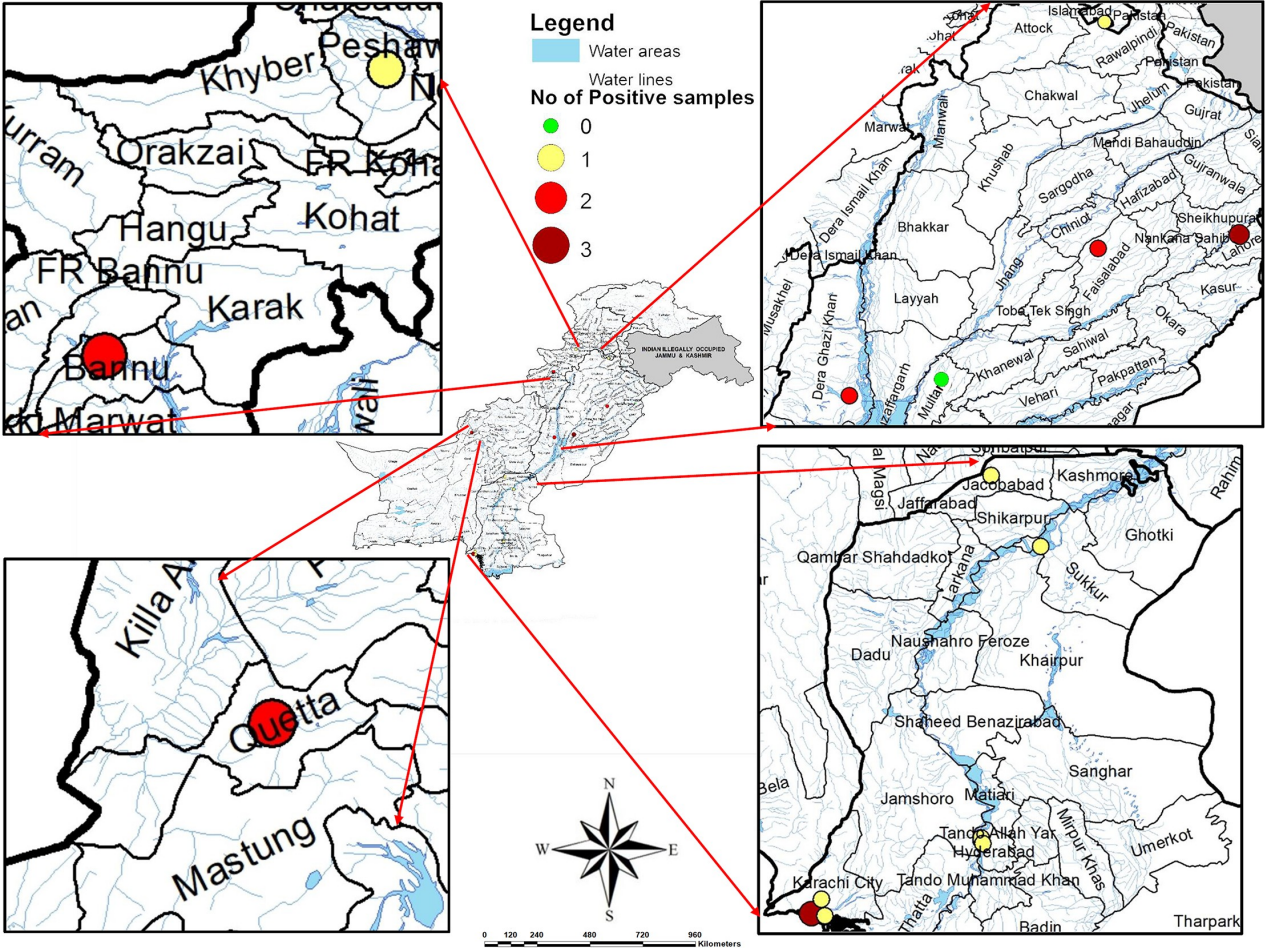
Map indicated environmental sites from where sewage water is collected and showing the geographic distribution from where cholera is tested in Pakistan, May 2019-Feb 2020.

There were 16 sites i.e. Safdarabad, Outfall-1, Pump Station-3uch Kaira, Ali Town, Makka Pumping Station, Tulsi Das Pumping Station, Qadir Nagar Pumping Station, Higrat Colony Pipc Nala, Hijrat Colongy Pidc Nala, Shoran Goth, Chakara Nala, Shaheen Muslin Town, Hinjal Noorbaz, Main Disposal DG Khan, Tausabad) take account of 12 districts (Bannu, Peshawar, Rawalpindi, Lahore, Faisalabad, Multan, DG Khan, Quetta, Karachi, Hyderabad, Jacobabad, and Sukkar) and covering four provinces of Pakistan i.e. KPK, Baluchistan, Punjab and Sindh (Fig 2). Selected sites were either open drains (10sites) or pumping stations (6sites) (Table 1) and were used for polio environment surveillance (ES). The total population subsidizing to wastewater at a given site ranged from <150,000 to >1,300,000 people, and the population under the age of five ranged from <20,000 to ~200,000 children [18].

**Table 1:** Geographic location, date, type of sampling sites and positive toxigenic *V*. *cholerae*.

District	Sample collection Date	Geographic location (Collection site)	Sample volume (L)	Type of sample site	Toxigenic *V*. *cholerae* positive samples
**Punjab**					
Rawalpindi	11-Oct-19	Safdarabad	6.00	Open drainage	1
Lahore	13-Jun-19	Outfall– 1	7.00	Pumping station	3
	18-Jul-19		6.00		
	18-Nov-19		7.00		
Faisalabad	7-Oct-19	Pump station-3 uch Kaira	6.00	Pumping station	2
	5-Nov-19		6.00		
Multan	-	Ali Town		Pumping station	-
DG Khan	12-Jul-19	Main Disposal DG Khan	6.00	Open drainage	2
	16-Dec-19		6.00		
**Sindh**					
Karachi	17-Sep-19	Hijrat Colony Pipc Nala	6.00	Open drainage	5
	23-Sep-19	Hijrat Colony Pidc Nala	6.50	Open drainage	
	9-Nov-19	Hijrat Colony Pidc Nala	6.00	Open drainage	
	12-Nov-19	Sohrab Goth	6.00	Open drainage	
	19-Nov-19	Chakara Nala	6.00	Open drainage	
Hyderabad	14-Nov-19	Qadir Nagar pumping station	6.60	Pumping station	2
	12-Nov-19	Tulsi Das Pumping Station	6.5	Pumping station	
Jacobabad	11-Jun-19	Saddar pumping station	6.00	Pumping station	1
Sukkar	5-Jul-19	Makka Pumping Station	6.00	Pumping station	1
**Balouchistan**					
Quetta	13-May-19	Tausabad	6.00	Open drainage	2
	12-Jun-19		6.00		
**KPK**					
Peshawar	10-Feb-20	Shaheen Muslim Town	6.0	Open drainage	1
Bannu	27-Jun-19	Hinjal Noorbaz	7.0	Open drainage	2
	29-Nov-19		7		

### Environmental factors

Monthly trends of *V*. *Cholerae* isolation from environmental samples were analyzed with respect to temperature and rainfall in order to find out environmental and temporal drivers associated with presence of *V*. *cholerae* in waste water. The average monthly temperature and rainfall during the period of the study were obtained from the regional meteorological centre. Seasonal trend was analysed by plotting frequency of toxigenic *V*. *cholerae* in every month of of study period (May 2019 to Feb 2020).

### Reference bacterial strain, media and culture

Reference strain of Vibrio spp. strain (NIH001V_C) used in this study were provided by Department of Microbiology, Shifa international hospital, Pakistan. The sample were initially incubated overnight at 30°C in an enrichment medium, consisting of 200 ml of alkaline peptone water (1% peptone, 1% Nacl) adjusted at pH = 8.6. After this a loop of the culture broth were taken from the top layer of alkaline peptone water and streaked on TCBS agar (Oxoid Ltd., England) and incubated overnight at 37°C. After appearance of yellow colonies on selective medium 6 to 12 flat colonies having 1–2 mm diameter were picked and subcultured onto nutrient broth in preparation for serovar confirmation and identification through API 20E (Biomérieux, Lyon, France) and VITEK® as per user recommendations with the manufacturer guidelines. Biochemical tests i.e. oxidase and motility test were also performed to confirm the *V*. *Cholerae* reference isolate [9].

### Bag-Mediated Filtration System (BMFS) samples

BMFS samples were collected in sampling bag, covered with a pore size (249μm) pre-screenmesh over the opening [26]. Onsite-filtration of samples has been done at 16 sites. Volume of wastewater samples from different sites are mentioned in Table 1. However, some of the samples were not be filtered onsite due to security apprehensions at the Quetta site, that’s why a “bucket” protocol was used to in transportation of samples to a secure locality. For this before sample collection, an insulated *Kool BucketTM* (Kool Buckets LLC, Huntington Station, NY, USA) was prepared by placing two pre-frozen cold packs. After that samples were collected and bag was closed with a sanitized (70% ethanol) clip and secure tight and placed into the insulated bucket for secondary containment in the course of travel to a secure a suitable locality.

Consequently all samples carry-over for the filtration, collection bag was hung on to a customized tripod (BoundaryTEC, Minneapolis, MN, USA). An encapsulated ViroCapTM filter (Scientific Methods, Inc., Granger, IN, USA) was attached to the outlet port of bag and the sample filtered by gravity. The filter was enclosed within a custom-made, injection-molded and clear polycarbonate sump (Proto-labs, Maple Plain, MN, USA) with a commercially available *bullet-proof polypropylene lid* (Pentek, Inc., Upper Saddle River, NJ, USA) [18]. After the filtration of all samples, the filter was transported to National Institute of Health, Islamabad and received within 48 hours. To improve bacterial survival encapsulated ViroCap filters were then eluted with 100 mL 1.5% beef extract (BBLTM Beef Extract powder; Becton, Dickinson and Company, Sparks, MD, USA) and 0.05 M glycine (Merck, Darmstadt, Germany) solution at pH 9.5 [18]. The eluent was injected into the filter inlet and incubated for 30 minutes before recovering the eluate through the filter outlet using a peristaltic pump (Cole Parmer, Vernon Hills, IL, USA). The recovered eluate was pH-adjusted to 7.0–7.5. Samples were then shaken (200 rpm, 4˚C, overnight), centrifuged (6000 x g, 4˚C, 30 minutes), and the pellet was resuspended in a final volume of 10 mL phosphate-buffered saline, pH 7.4.

### Environmental sample collection and processing

Samples were collected using the Bag-Mediated Filtration System (BMFS) from each selected sites and processed as described previously [26]. Sampling personnel strictly followed the standard safety guidelines and used personnel protective equipment (PPE) required for waste water sampling. Following on-site filtration, the filters were brought to the laboratory in cold condition using frozen ice packs. Once in the laboratory, the microorganisms captured on the filter were eluted using beef extract solution. The eluate was processed by skim milk flocculation and the flocs were pelleted by centrifugation. The pellet obtained was re-suspended in 2 ml of phosphate buffered saline by using the QIAamp DNA Mini Kit. This DNA (5 μℓ) was intended to be used as template in a real-time PCR assay targeting the ctxAB [27]. Unionized water were used as negative controls in each of the experiments.

### Determination of specificity of PCR assay

*V*. *cholerae* was inoculated in Brain Heart Infusion (BHI) broth, and set aside at 37°C for 16–18 h. After growth 10-fold serial dilutions were made in PBS (pH was maintained at 7.4). Bacterial enumeration to determine CFU/mℓ was done by plating 100 μℓ of each dilution on BHI agar plates, followed by incubation at 37°C for 18 h. A 1mℓ culture from each dilution was washed twice with de-ionised water by centrifugation at 10000Xg for 10 min and finally re-suspended in 1 mℓ de-ionised water. 5 μℓ of each dilution was used directly as template in PCR.

### RT-PCR detection of ctxAB positive toxigenic *V*. *cholerae* from environment samples

The qPCR cycling protocol and reaction component concentrations were optimized for detection of the ctxAB gene. The primers used were ctxAB-F (5’AGGGAAGAGCCGTGGAT-3’), ctxAB-R (5-ACTTTGGGTTTTTTCATCGCAAG-3’) and a probe within the ctxAB gene (5-FAM-CATCATGCACCGCCGGGTTGTG-BHQ-3) labelled with 5′ labelled with FAM and a Black Hole Quencher 1(BHQ1) on the 3′ end (Integrated DNA Technologies) to detect the exogenous internal amplification control (IAC) used for amplification to detect *V*. *cholerae* were reported previously [27]. Primers were selected from previous studies and were designed on the basis of GenBank gene sequences of *V*. *cholerae* using Oligo Explorer version 1.2 (Gene Link). Amplifications were carried out in real time PCR thermal cycler (Applied Bio Systems, 7500 US) automated for thirty three-step cycles started with denaturation of the template DNA at 95°C for 45 s; annealing at 56°C for 45 s and extension of the primers at 72°C for 1 min. Before initiation of the first cycle, the reaction mixture was heated at 94°C for 10 min to allow complete denaturation of the template. After the last cycle, the reaction mixture was subjected to 72°C for 10 min to ensure final extension. In a control PCR reaction, deionised water was added to the reaction mixture instead of bacterial cells.

### Data management and analysis

Each wastewater sample was assigned a unique specimen identification (SID) number immediately after collection in the field with the help of BFMS method. This number was used to identify the samples and accompanying form that went to laboratory with the samples. From the data on the paper forms, results were entered into a database using the IBM® SPSS® statistical package. GIS coordinates were collected, cleaned, and stored in spreadsheet. Double entry and data cleaning was done to remove errors generated in the process data collection and entry. Analysis was done to get frequencies and percentages. The maps were created using Arc View Geographical Information Software3 (Arc GIS). Significance of cycle threshold value (CT value) were estimated in univariate analysis with 95% confidential intervals by using t-test, p < 0.05 was taken as significant for CT value of cholera load in waste water within the selected sites across the four provinces of country.

## Results

**Fig 3:**
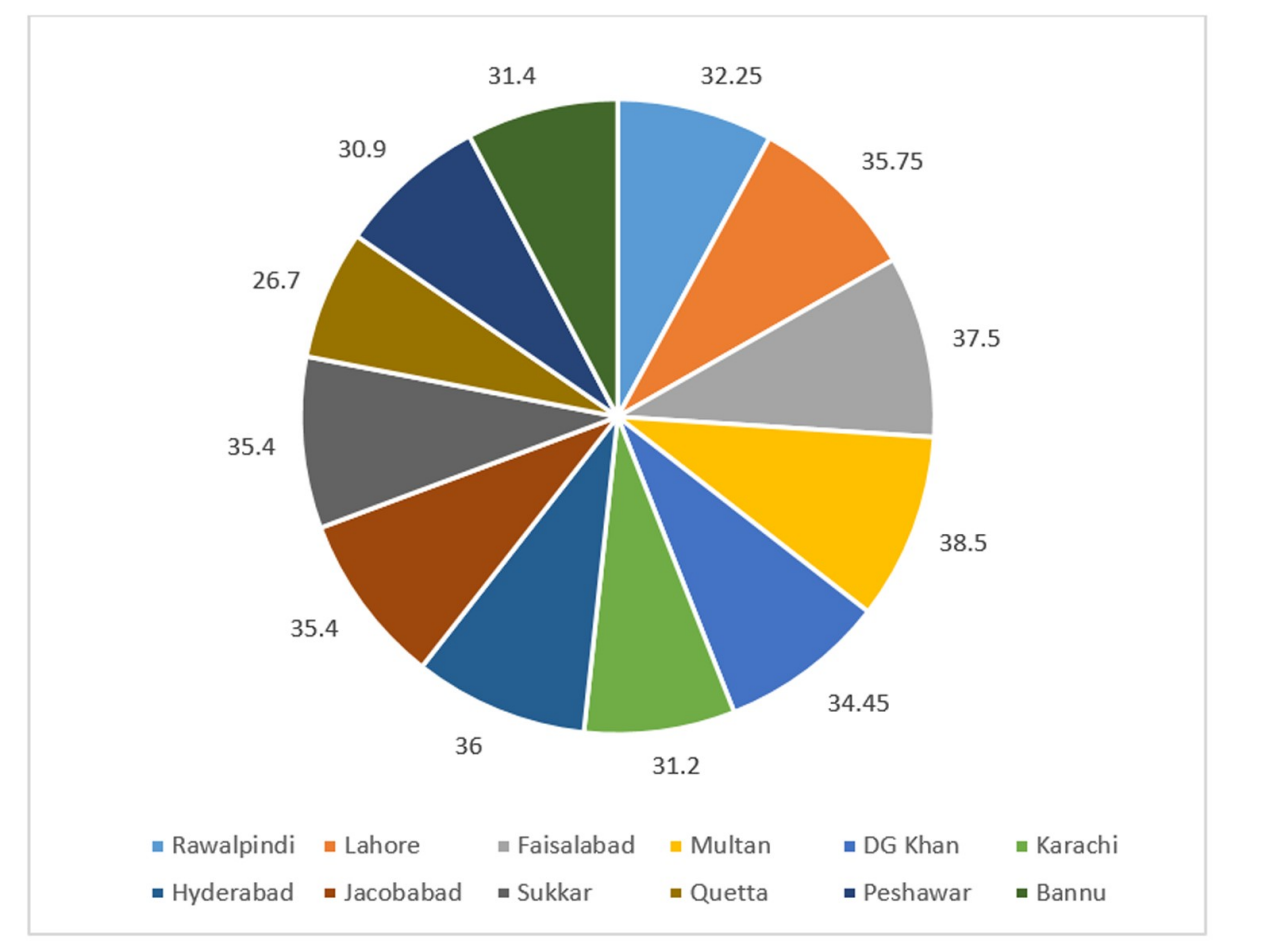
Average temperature in various regions of Pakistan during 2019.

**Fig 4:**
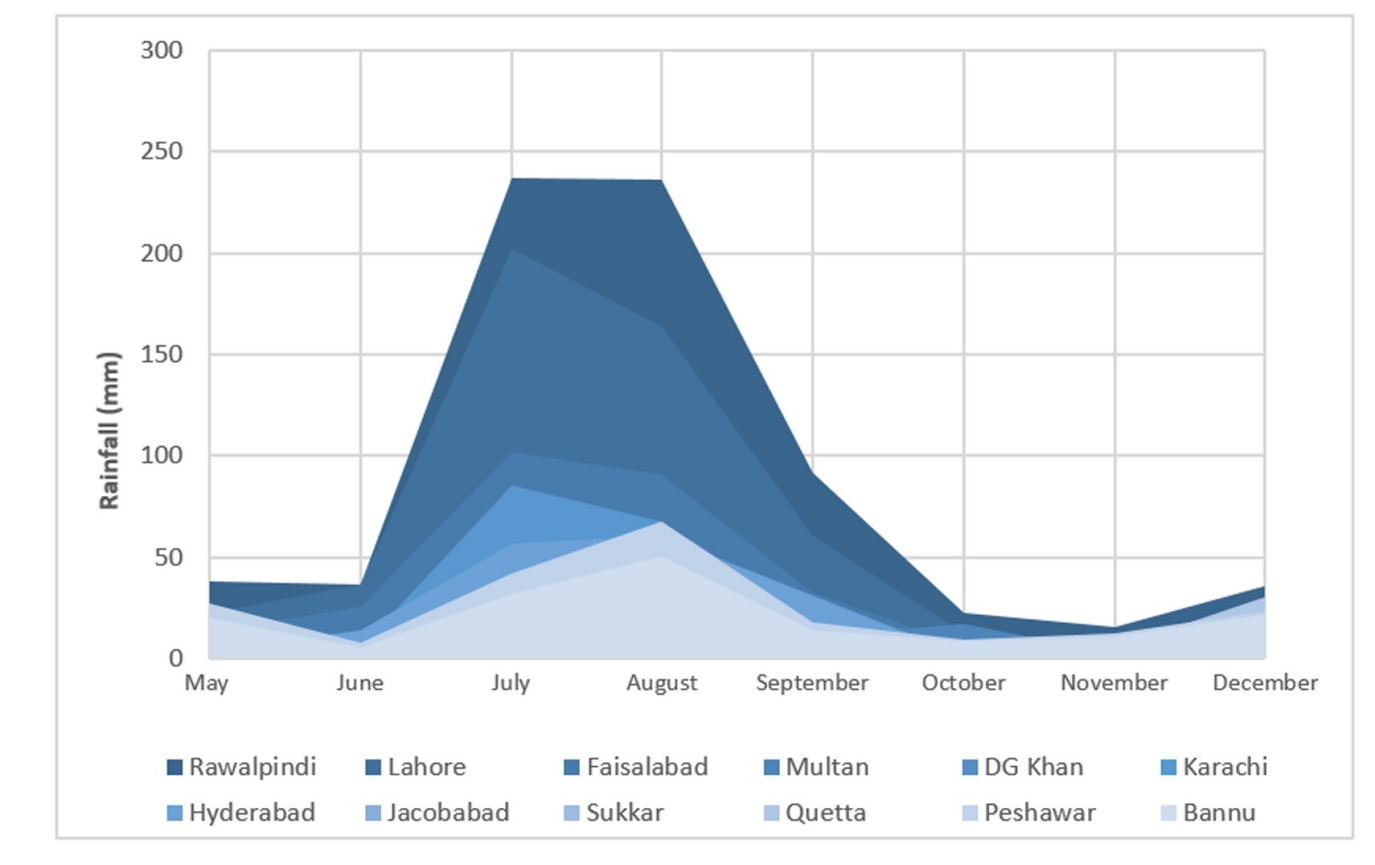
Average rainfall in various regions of Pakistan during 2019.

**Fig 5:**
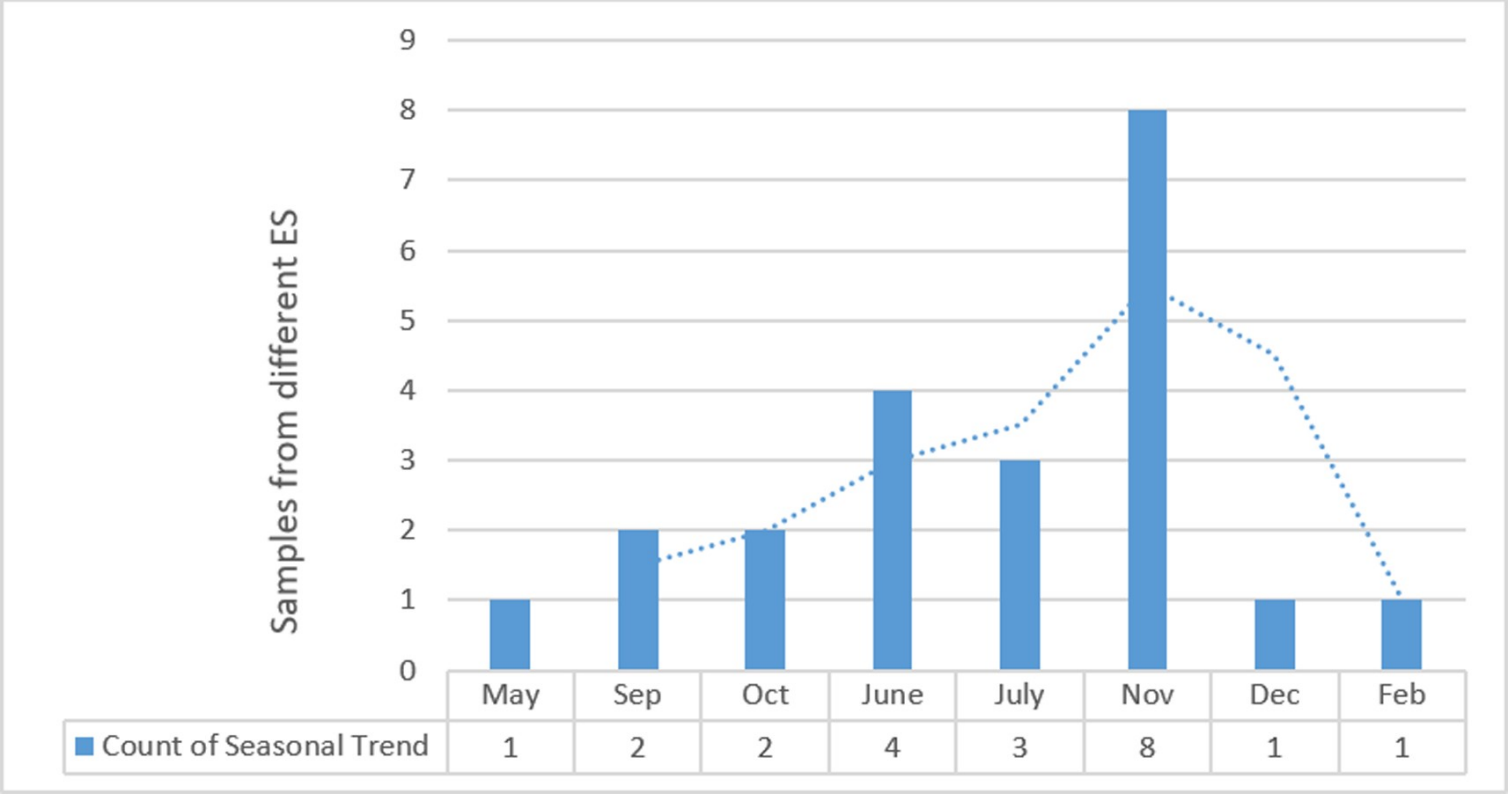
Seasonal trend of *Vibrio cholera* in waste water collected from May 2019- Feb 2020. Each month containing mean of isolations detected from different environmental sites across the Pakistan.

The current study was aimed to examine the environmental surveillance of *V*. *Cholera* in Pakistan. The description of sample collection from various regions is represented in Table 1. Out of 160 environmental samples, ctxAB qPCR fluorogenic probe and primer set were tested for detection of toxigenic *V*. *cholerae* in waste water. RT-PCR result showed 22 positive samples against cholera toxigenic gene, mean (**34±2**). Sites that are from Karachi district i.e. Hijrat Colony Pidc Nala and Sohrab Goth and Chakara Nala showed lowest CT values with significant difference (p < 0.05) i.e. 32 and 34 respectively. This indicates higher *V*. *cholerae* load in Karachi district that is eventually very alarming situation for periodic cholera outbreak. The average monthly temperature and rainfall during the period of the study in relation to isolation of *V*. *cholera* from environment sources is shown in Figs 3 and 4. The highest average temperature recorded was 38.5°C in Multan while the highest average rainfall was 82.7 mm in Rawalpindi as represented in Figs 2 and 3. The highest average rainfall was observed in Rawalpindi, followed by Lahore and Peshawar. Similarly, 3 samples from Rawalpindi were positive while one sample from Lahore were detected positive. The highest number of positive sample was 5 in number which were collected from Karachi. Although the average rainfall and temperature in Karachi are reported normal, however, the poor sanitation problems could be attributed for highest environmental surveillance of V. cholera. The isolation of *V*. *cholera* from the environmental sample in relation to rainfall and temperature has been represented. The seasonal trend of *V*. *cholera* detection in waste water of different regions is represented in Fig 5. Seasonal trend showed maximum positive samples were reported in month of november. Samples from Multan district doesn’t show positive sample, reason can be high temperature that is showing in Fig 3. Further analysis of test results indicated that wastewater samples collected from quetta had the had the highest percentage positive at 20%. However, only a few wastewater samples were collected (Table 2).

**Table 2:** Percentage distribution of *Vibrio cholerae* in wastewater by districts in Pakistan during the study period, May 2019- Feb 2020.

Province	District	Total Sites	Total sample collected	No. positive samples	Percentage
**Punjab**	5	5	50	8	16%
**Sindh**	4	8	80	9	11%
**Balochistan**	1	1	10	2	20%
**KPK**	2	2	20	3	15%

## Discussion

Cholera is a communicable infectious disease, due to its epidemiologic behaviour it has a tendency to occur periodically in explosive outbreaks especially in developing countries and present a threat to the public health [28]. It was originated from Asian subcontinent long ago and was responsible for several severe outbreak around the globe. Being a developing country, Pakistan is facing a burden of several infectious diseases contributing to more than 26% of the total diseases burden. The major infectious diseases in Pakistan includes acute respiratory diseases, Typhoid, Malaria, viral hepatitis, dengue and cholera [29].Cholera outbreaks are escalating steadily, and country has been experiencing intervals of sporadic cases and localized outbreaks in the last few years. Pakistan belongs to northern hemisphere and follows two seasonal trend of cholera according to a study conducted previously [15]; one is December through May and season two is June through November. In our study frequency of *V*. *cholerae* in waste water ranges from May to December where peak were seen in November (27^0^ C) from Sindh province then in June from different districts. Temperature and rainfall may influence the temporal fluctuations of cholera, potentially will increase the frequency and duration of cholera across the country. Number of cholera positive samples were high in Sindh relatively in month of September, may be because of high temperature and poor sanitation system of Sindh province. Moreover, in Karachi generally receives monsoon rains from July to September, and there has not been a significant variation in terms of the timeframe of the monsoon season in Karachi over the past 25 years. However, a key characteristic of Karachi’s climate is the often unpredictable level of rainfall on a yearly basis. Seasonal deviations in monsoon rainfall and associated flooding affect the severity and distribution of *V*. *cholera* [30,31]. Likewise, in 2020, Cholera outbreak reported in Quetta District, Balochistan Province which also caused mortality [19]. Moreover, according to reports, 62 percent of Balochistan is deprived of safe drinking water and lack of access to basic sanitation facilities. This is another dynamic that stimulate the peril which is another alarming situation for a country. In addition to this, *V*. *cholera* detection also do not follow a clear seasonal pattern, as well as its presence in waste water depict that it can be reservoir of future cholera epidemic. Current study provides an estimate about the burden of *V*. *cholerae* in environment. Cholera environmental surveillance system has been established by integration in existing country’s polio environmental surveillance network. Bag-Mediated Filtration system, a separation method were employed for collection of effective sampling by utilizing World Health organization (WHO) protocols [32] to achieve the great and effective volume of processed waste water samples. Further screening of toxigenic *V*. *cholerae* has been done by utilizing molecular based detection to assess the burden of Cholera. The outbreaks of cholera in Pakistan could be attributed to the poor sanitation/environmental condition as seen in typhoid outbreaks in Hyderabad [33], and also usage of contaminated water or consumption of unhygienic/contaminated food [34]. In this study high frequency of toxigenic cholera has been showed in Karachi city waste water, and it has been speculated that as Karachi is a metropolitical city where there is poor sanitation system [35]. It has been previously reported that *V*. *cholerae* in the environmental samples could be linked with the localized outbreaks of acute watery diarrhoea [36]. Its presence also signifies several outbreaks of diarrhoea due to other bacterial and non-bacterial infections [37,38]. The *V*. *cholerae* has also been reported to acquire pathogenicity and virulence factors encoded in the mobile genetic elements from the surrounding [39]. As the *V*. *cholerae* was present in the environment therefore the emergence of virulent strains and consequent outbreaks could be expected. There is a need of further exploration of the topic to study other characteristics of the sample source and to guide the surveillance system to monitor and take preventive measure of such threats on time.

### Strength of this study

The strength of this study lies in the following facts: Firstly, the investigators purposively selected routine polio surveillance environmental sites to explore the integrative approach for environmental surveillance of *V*. *cholerae* in wastewater. This purposive selection of wastewater sites would result in detection of toxigenic (epidemic) *V*. *cholerae*. Secondly, the fact that the test methods were frequently suitable to detect toxigenic *V*. *cholerae* [40]. Third, detection methods that were used were also found efficient in other studies for detection of Vibrios species. [41,42]. In addition to this, there was no evidence to support the wastewater source serving as reservoir for epidemic *V*. *cholerae* is also supported by both epidemiologic and molecular studies on spread of cholera and genetic relatedness of *V*. *cholerae* bacteria responsible for cholera outbreaks in Pakistan [43,44]. Environmental surveillance methods to detect *V*. *cholerae* in water will enable low middle income class country (LMIC) like Pakistan to estimate cholerae transmission in their communities and enable rational decisions in cholera vaccination campaigns. However, there is very less or no surveillance and research base data of cholera in Pakistan. This study provides a future threat of cholera outbreak.

### Limitation of this study

The study involved testing of wastewater samples from 12 districts, it is possible that the reservoirs could be in other regions as well which were not considered in study, and that could be a major threat of future cholera outbreaks in Pakistan. Therefore, further studies using appropriate method and targeting other regions that is not covered by our study should be conducted to provide more information on *V*. *cholerae* in wastewater sources that is circulating in water bodies and contaminated drinking water. Furthermore, this study was conducted approximately for about period of 1 year; possibly the results might have been different if it were to continue for more years; however, we believe that this time was adequate for us to establish the presence of *V*. *cholerae* in a cholera endemic regions.

### Conclusion

From our study, it could be concluded that *V*. *cholerae* in waste water could be alarming for a potential cholera outbreaks in the country. Preventive measure need to be taken against an alarming intensification of infections in near future. The central and local government should take proper consideration and identify the higher-risk areas within Pakistan. This will serve in planning or monitoring the risk factors followed by adaptation of precautionary measure precedent in risk areas and will ultimately help countries in cholera elimination goal 2030.

*V*. *cholerae* detection do not follow a clear seasonal pattern, presence of V. cholera in waste water shows that it can be reservoir because of which cholera is prevalent or circulating round the year across the Pakistan. There is no proper cholera surveillance system in the country due to low resources and poor management. Only few selected areas have small number of comprehensive surveillance system. This is the very first study which is contributing towards the detection of environmental surveillance of cholera across the Pakistan. However pragmatic studies should have been done to rigorously examine the temporal variation and seasonality of cholera. This will eventually help in understanding of sporadic cholera outbreak and control the liability of this global syndrome. This study can be used to improve the surveillance of toxigenic *V*. *cholerae* in Pakistan, by developing a separate lab-based molecular surveillance system using the already established current polio surveillance system.
